# *Botrytis cinerea* BcCDI1 protein triggers both plant cell death and immune response

**DOI:** 10.3389/fpls.2023.1136463

**Published:** 2023-04-25

**Authors:** Wenjun Zhu, Huange Dong, Ran Xu, Jingmao You, Da-zhong Yan, Chao Xiong, Jing Wu, Kai Bi

**Affiliations:** ^1^ School of Life Science and Technology, Wuhan Polytechnic University, Wuhan, China; ^2^ Key Laboratory of Biology and Cultivation of Chinese Herbal Medicines, Ministry of Agriculture and Rural Affairs, Enshi, China; ^3^ Institute of Chinese Herbal Medicines, Hubei Academy of Agricultural Sciences, Enshi, China

**Keywords:** CDIPs, *Botrytis cinerea*, BcCDI1, elicitor, plant defense

## Abstract

Cell death-inducing proteins (CDIPs) play important roles in the infection of *Botrytis cinerea*, a broad host-range necrotrophic phytopathogen. Here, we show that the secreted protein BcCDI1 (Cell Death Inducing 1) can cause necrosis in tobacco leaves and at the same time elicit plant defense. The transcription of *Bccdi1* was induced at the infection stage. Deletion or overexpression of *Bccdi1* resulted in no notable change in disease lesion on bean, tobacco, and *Arabidopsis* leaves, indicating that *Bccdi1* has no effect on the final outcome of *B. cinerea* infection. Furthermore, the plant receptor-like kinases BAK1 and SOBIR1 are required to transduce the cell death-promoting signal induced by BcCDI1. These findings suggest that BcCDI1 is possibly recognized by plant receptors and then induces plant cell death.

## Introduction

*Botrytis cinerea* is a necrotrophic fungus and typical broad host-range phytopathogen, which leads to gray mold and rot diseases in a wide range of agriculturally important crops, causing severe economic loss worldwide (Dean et al., 2012; Veloso and van Kan, 2018). To date, *B. cinerea* has been used as a model fungus for studying the interaction mechanism between necrotrophic pathogens and plants. However, the molecular mechanism for the pathogenicity of *B. cinerea* remains largely unknown.

Plants are constantly confronted with various microbial pathogens, some of which are devastating in agriculture. Plants can survive due to the development of sophisticated defense systems during the long-term coexistence with phytopathogens (Jones and Dangl, 2006). The first defense layer of plants is pathogen-associated molecular pattern (PAMP)-triggered immunity (PTI), in which the host receptors recognize the PAMPs of various pathogens and induce effective resistance responses against them. Some recent studies have demonstrated that plants can employ multiple cell surface pattern recognition receptors (PRRs) to sense and recognize the PAMPs of diverse pathogens, thereby initiating a series of intracellular signaling and then activating various immune defense responses against the pathogen (Schellenberger et al., 2019; Albert et al., 2020; Zhou and Zhang, 2020). In addition, many studies have also revealed that several of these receptors can interact with the leucine-rich repeat (LRR) receptor-like kinase (RLK) BRI1-ASSOCIATED KINASE-1 (BAK1) and SUPPRESSOR OF BIR1-1 (SOBIR1) to form signaling-competent receptor complexes required for immune signaling (van der Burgh et al., 2019).

For overcoming plant immunity and effectively infecting the host, biotrophic and hemibiotrophic pathogens generally secrete diverse effectors to the interaction area between fungal hyphae and the host or into plant cells during infection to prevent host detection or interfere with the plant defense system (Stergiopoulos and de Wit, 2009; Lo Presti et al., 2015; Kim et al., 2016). Plants have also co-evolved the effector-triggered immunity (ETI) mediated by Resistance (R) proteins, which is often associated with the hypersensitive response (HR) of local plant cells at the infection site, thereby restricting the further progression of infection by biotrophic and hemibiotrophic pathogens (Jones and Dangl, 2006; van Ooijen et al., 2007; Cui et al., 2015). However, HR and the related programmed cell death (PCD) of plants are ineffective for necrotrophic pathogens, and necrotrophs can even exploit the HR and PCD to facilitate their infection. For example, Victorin, an effector protein secreted by the host-specific necrotroph *Cochliobolus victoriae*, can elicit a resistance-like response in *Arabidopsis thaliana* and then facilitate disease progress (Lorang et al., 2007; Lorang et al., 2012). Other two host-specific necrotrophic wheat pathogens, *Parastagonospora * and *Pyrenophora tritici-repentis*, can secrete multiple necrotrophic effectors to induce severe necrosis and confer disease susceptibility in wheat genotypes harboring specific sensitivity genes (Oliver et al., 2012; Gao et al., 2015; Shi et al., 2015; Dagvadorj et al., 2022).

Eizner et al. (2017) reported that the infection process of *B. cinerea* on the host plant typically comprises three stages: the early stage characterized by the formation of local necrosis lesions, the intermediate stage determining the outcome of *B. cinerea* infection, and the late stage of fast lesion spreading. Since *B. cinerea* achieves its infection and colonization by killing plant cells, it secretes diverse effector proteins to manipulate the host defense and/or induce necrosis to facilitate its infection on the host plants (Heard et al., 2015; Denton-Giles et al., 2020; Bi et al., 2021; Choquer et al., 2021; Reboledo et al., 2021; Shao et al., 2021; Zhu et al., 2022). These secreted effector proteins play important roles in the pathogen–plant interaction. However, little is known about the biochemical activities and molecular mechanisms for the effect of such necrotrophic effectors.

In this study, to analyze potential *B. cinerea* effectors, we further identified and characterized a secreted protein BcCDI1 with cell death-inducing activity reported by Franco-Orozco et al. (2017). Our results demonstrated that BcCDI1 could activate the defense response of *Nicotiana benthamiana* and its necrosis-inducing signal is mediated by BAK1 and SOBIR1.

## Materials and methods

### Fungi, bacteria, plants, and culture conditions

The *B. cinerea* wild-type strain B05.10 and the derived transformants were grown and maintained on PDA medium (Acumedia, MI, USA) at 22°C under continuous fluorescent light supplemented with near-UV (black) light. The conidia of all *B. cinerea* strains were obtained after 7 days of culture. The *Escherichia coli* strains of BL21 (DE3) and DH5α (Shanghai Weidi Biotechnology, Shanghai, China) were respectively used for protein expression and plasmid construction. The *Agrobacterium tumefaciens* strain GV3101 (Shanghai Weidi Biotechnology, Shanghai, China) was used for *Agrobacterium*-mediated transient expression of target proteins in plant leaves.

*Arabidopsis thaliana* (Columbia-0) was grown in a greenhouse under 16-h/8-h intervals of light/dark at 22°C/20°C. Tobacco (*N. benthamiana*), wheat (*Triticum aestivum* cv. Galil), bean (*Phaseolus vulgaris*, genotype N9059), tomato (*Solanum lycopersicum* cv. Hawaii 7981), and maize (*Zea mays* cv. Silver Queen) plants were grown in a greenhouse under 16-h/8-h intervals of light/dark at 25°C/22°C.

### Plasmid construction

The *Bccdi1* deletion construct was generated as described previously (Zhu et al., 2017). The 5′ (550 bp) and 3′ (551 bp) flanking fragments of the *Bccdi1* gene were amplified and respectively cloned into the upstream and downstream regions of the *hph* cassette using Gibson Assembly Master Mix kit (New England Biolabs, MA, USA). The overexpression plasmid was generated by cloning the full-length open reading frame of *Bccdi1* into the pH2G vector under the regulation of *B. cinerea* histone H2B promoter (NCBI identifier CP009806.1) and the endo-β-1,4-glucanase precursor terminator (NCBI identifier CP009807.1) as described previously (Zhu et al., 2017).

For transient expression of the target protein in plants using the agroinfiltration method, the indicated sequences fused with the 3×FLAG tag were cloned into vector pCHF3 between the 2×CaMV 35S promoter and the NOS terminator and then transformed into the *A. tumefaciens* strain GV3101. The *E. coli* protein expression vector was constructed by cloning the *Bccdi1* mature sequence without the signal peptide (BcCI1^ΔSP^) into the vector pTac-His-MBP-PreScission (Beyotime Biotechnology, Shanghai, China) and then transformed into the *E. coli* strain BL21 (DE3).

### Manipulation of nucleic acids

The total RNA of fungi and plants was isolated using the RN03-RNApure Kit (Aidlab, Beijing, China), and the residual DNA in RNA was removed using DNase I (Thermo Scientific, MA, USA) according to protocols and stored at −80°C. The first-strand cDNA of the samples was generated using the cDNA synthesis kit (Simgen, Hangzhou, China). Reverse transcription-quantitative PCR (RT-qPCR) was performed to analyze the gene expression profile using SYBR^®^ Green Mixes (Simgen, Hangzhou, China) and CFX96 Touch Real-Time PCR Detection System (Bio-Rad, CA, USA) according to the manufacturer’s instructions. The *B. cinerea Bcgpdh* gene and the *N. benthamiana NbEF1α* gene were respectively used as endogenous control genes to normalize the expression level of target genes as described previously (Zhu et al., 2017). For each analyzed gene, the RT-qPCR assay was repeated at least twice, and each repetition included three independent replicates. The genomic DNA of the indicated *B. cinerea* strains was isolated using the Fungal Genomic DNA Purification Kit (Simgen, Hangzhou, China) according to the protocol. All the primers used are listed in **Supplementary Table S1**.

### Bioinformatics analysis

The NCBI (http://www.ncbi.nlm.nih.gov/) database was used to obtain the homologous sequences of BcCDI1 in other pathogens through a BLASTp analysis. The JGI (http://genome.jgi.doe.gov/Botci1/Botci1.home.html) database of *B. cinerea* was used to characterize *B. cinerea* genomic and transcriptomic sequences. The SMART (http://smart.embl-heidelberg.de/smart/change_mode.pl) was used to analyze the protein domain and predict signal peptide sequence. The Clustal X and MEGA-X programs were used for protein alignments and phylogenetic tree construction with an unrooted neighbor-joining method.

### Transformation, pathogenicity, and cell wall stress tolerance assay of *Botrytis cinerea*

Genetic transformation of *B. cinerea* was performed as described previously (Ma et al., 2017). The *Bccdi1* deletion mutant *ΔBcCDI1* and the overexpression strain OEBcCDI1 were confirmed using PCR and RT-qPCR.

Pathogenicity assay on the primary leaves of 9-day-old bean or tobacco was performed as previously described (Zhu et al., 2017). The conidia of the indicated *B. cinerea* strains were suspended in an inoculation medium [Gamborg’s B5 medium containing 2% (w/v) glucose and 10 mM of KH_2_PO_4_/K_2_HPO_4_, pH 6.4]. Then, 8 μl of conidial suspension (1 × 10^5^ conidia/ml) was inoculated on the leaves of the bean or tobacco, or the mycelial plugs cut from the edge of *B. cinerea* were inoculated on the leaves of the plants. The infected plants were incubated in a humid chamber at 22°C for 48 or 72 h, and the lesion diameter was then measured.

To analyze the possible effect of BcCDI1 on the cell wall integrity of *B. cinerea*, the indicated strains were inoculated on PDA plates supplemented with 0.5 mg/ml of Congo Red, 0.3 mg/ml of Calcofluor White, 1 M of sorbitol, 1 M of NaCl, and 0.02% SDS at 22°C, as described previously (Zhu et al., 2022).

### *Agrobacterium tumefaciens*-mediated transient expression and Western blotting assay

*Agrobacterium tumefaciens*-mediated transient expression in *N. benthamiana* leaves was performed using the agroinfiltration method as previously described (Kettles et al., 2017). Extraction of the total protein of plants or fungi was performed. Approximately 0.2 g of tissue was ground to a powder using liquid nitrogen and suspended in 1 ml of cold lysis buffer (Beyotime Biotechnology, Shanghai, China). Then, the target samples were incubated on ice for 5 min and centrifuged at 12,500 rpm for 10 min at 4°C to collect the supernatant-soluble proteins. The supernatant proteins were then mixed with 5× SDS-PAGE sample buffer (Beyotime Biotechnology, Shanghai, China) and denatured by boiling for 10 min at 100°C. The denatured proteins were separated by SDS-PAGE electrophoresis and transferred onto PVDF membranes (0.45 μm). Western blotting was carried out using an anti-GFP antibody (Beyotime Biotechnology, Shanghai, China).

### Expression and purification of recombinant His-MBP-BcCDI1^ΔSP^ protein

The expression of His-MBP-BcCDI1^ΔSP^ and His-MBP-GFP recombinant proteins was performed in the *E. coli* strain BL21 (DE3) as previously described (Zhu et al., 2017). Purification of recombinant proteins was performed using Glutathione Beads 4FF (Smart-Lifesciences, Changzhou, China) following the manufacturer’s instructions. The proteins were cleaned using Amicon Ultra-4 Centrifugal Filter Devices (15 ml, 10 kDa; Merck Millipore, Darmstadt, Germany) to remove the elution buffer, then dissolved in phosphate-buffered saline (PBS), and stored at –80°C.

### Protein infiltration assay and induction of plant resistance by BcCDI1

To test the necrosis-inducing activity of BcCDI1 in plants, 100 μg/ml of recombinant protein produced in *E. coli* was infiltrated into the plant leaves using a syringe. The plants were then kept in a greenhouse at 25°C and photographed 5 days post-infiltration.

To test the plant resistance-inducing activity of BcCDI1, *N. benthamiana* leaves were infiltrated with 5 μg/ml of the tested protein. The infiltrated plants were kept in the greenhouse for 2 days, and the treated leaves were then inoculated with *B. cinerea* for an additional 2 days or used for the expression analysis of defense-related genes.

### VIGS in *Nicotiana benthamiana*

To determine whether BcCDI1-induced plant death is associated with *NbBAK1* or *NbSOBIR1* in tobacco, virus-induced gene silencing (VIGS) was performed to silence the expression of *NbBAK1* or *NbSOBIR1* as previously described (Zhu et al., 2017). The pTRV2::*GFP* plasmid was used as the control. The expression level of *NbBAK1* or *NbSOBIR1* in gene-silenced *N. benthamiana* was determined by RT-qPCR analysis. Then, *Bccdi1* was expressed through *A. tumefaciens*-mediated transient expression in the leaves of *NbBAK1-* or *NbSOBIR1*-silenced *N. benthamiana*. The infiltrated plants were then kept in the greenhouse at 25°C, and the necrosis development was photographed 5 days after treatment.

## Results

### BcCDI1 contains a hypothetical signal peptide without a known domain

*Bccdi1* (*BCIN06g00550*) is a single-copy gene encoding 202 amino acids in *B. cinerea*. Bioinformatics analysis revealed that the first 19 N-terminal amino acids are the potential signal peptide and there is no transmembrane domain, indicating that BcCDI1 may be a hypothetical secreted protein. In addition, *SMART MODE* analysis identified no protein domain or possible function in the BcCDI1 sequence. BLAST search against the NCBI database with the BcCDI1 sequence demonstrated that all homologs of BcCDI1 are only present in Ascomycete fungi and were not found in other phyla of fungi or in bacteria or oomycetes. All BcCDI1 homologs are annotated as hypothetical proteins. Multiple sequence alignment and phylogenetic analysis demonstrated significant sequence similarity between BcCDI1 and its homologs (**Supplementary Figure S1**).

### BcCDI1 is a secreted protein with necrosis-inducing activity

**Figure 1:**
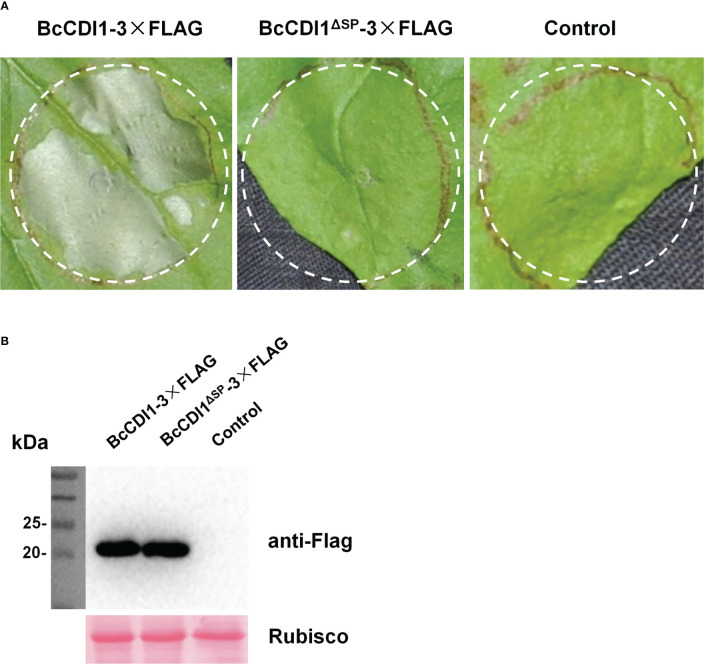
Full-length BcCDI1 can induce cell death, and the signal peptide is required for its function. **(A)** Images of *Nicotiana benthamiana* leaves at 5 days after agroinfiltration with *Agrobacterium tumefaciens* strains carrying the indicated constructs. *Agrobacterium tumefaciens* carrying the empty vector was used as the control. BcCDI1-3×Flag, BcCDI1 fused with 3×Flag at the C-terminus; BcCDI1^ΔSP^-3×Flag, BcCDI1 without the signal peptide fused with 3×Flag at the C-terminus. **(B)** Immunoblot analysis of proteins from *N. benthamiana* leaves with transient expression of the indicated constructs. Top panel, immunoblot using anti-Flag antibody; bottom panel, staining of the Rubisco large subunit with Ponceau S.

*Agrobacterium tumefaciens*-mediated transient expression (agroinfiltration) was used to test the necrosis-inducing activity of candidate effector proteins in *N. benthamiana* leaves. The transient expression of full-length BcCDI1 could cause necrosis in *N. benthamiana* leaves (**Figure 1**), whereas that of the control plasmid or BcCDI1^ΔSP^ without the predicted secretion signal peptide did not trigger leaf death (**Figure 1**). Since the function of the signal peptide is to guide the protein into the plant apoplastic space after transient expression in plant cells and the first 19 N-terminal amino acids are predicted to be the signal peptide, it could be inferred that BcCDI1 may be a hypothetical secreted protein that functions in the leaf apoplastic space to induce plant cell death under the guidance of the signal peptide.

**Figure 2:**
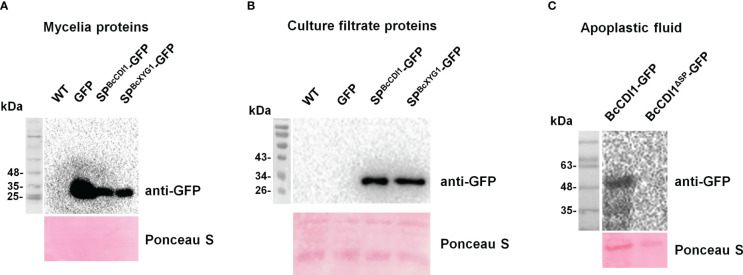
BcCDI1 signal peptide possesses secretion function. **(A)** Immunoblot analysis of the total mycelial proteins from the indicated *Botrytis cinerea* strains. Top panel, immunoblot using anti-GFP antibody; bottom panel, Ponceau S staining of the total mycelial proteins. **(B)** Immunoblot analysis of culture filtrate proteins from the indicated *B cinerea* strains. Top panel, immunoblot using anti-GFP antibody; bottom panel, Ponceau S staining of the secreted proteins. SP^BcCDI1^-GFP, GFP fused with the signal peptide of BcCDI1; SP^BcXYG1^-GFP, GFP fused with the signal peptide of BcXYG1; GFP, GFP overexpression strain; WT, wild-type strain. **(C)** Western blotting analysis of apoplastic fluid from *Nicotiana benthamiana* leaves agroinfiltrated with BcCDI1-GFP or BcCDI1^ΔSP^-GFP for 3 days. Top panel, immunoblot using anti-GFP antibody; bottom panel, Ponceau S staining of the apoplastic fluid.

Two methods were further used to confirm whether BcCDI1 is indeed a secreted protein. First, the signal peptide of BcCDI1 was fused with GFP to generate SP^BcCDI1^-GFP. Similarly, we fused the signal peptide of BcXYG1, a strong cell death-inducing secreted protein of *B. cinerea* that functions in the apoplastic space (Zhu et al., 2017), with GFP to generate SP^BcXYG1^-GFP as the positive control, and the GFP overexpression construct without the signal peptide was used as the negative control. All the constructs were transformed into the wild-type *B. cinerea* strain to generate overexpression strains of the fused proteins. Western blot analysis showed that all the indicated proteins were successfully expressed in the hyphae of each strain (**Figure 2A**). Then, all examined strains were respectively cultured in potato dextrose broth (PDB) liquid medium for 3 days, and an immunological analysis was performed to check the presence of GFP in the culture filtrate. The results confirmed the accumulation of GFP in the culture medium of SP^BcCDI1^-GFP and SP^BcXYG1^-GFP overexpression strains, but not in that of GFP overexpression or wild-type strains (**Figure 2B**). For the second method, BcCDI1 and BcCDI1^ΔSP^ were fused with GFP to generate transient expression plasmids of BcCDI1-GFP and BcCDI1^ΔSP^-GFP. Within 2 days after agroinfiltration, Western blot analysis successfully detected BcCDI1-GFP but not BcCDI1^ΔSP^-GFP in apoplastic fluid from infiltrated tobacco leaves (**Figure 2C**), indicating that BcCDI1-GFP was indeed secreted into the apoplastic space of plant cells. In summary, all these results confirmed that BcCDI1 is a secreted protein.

### BcCDI1^ΔSP^ induces cell death in tobacco, tomato, and potato

**Figure 3:**
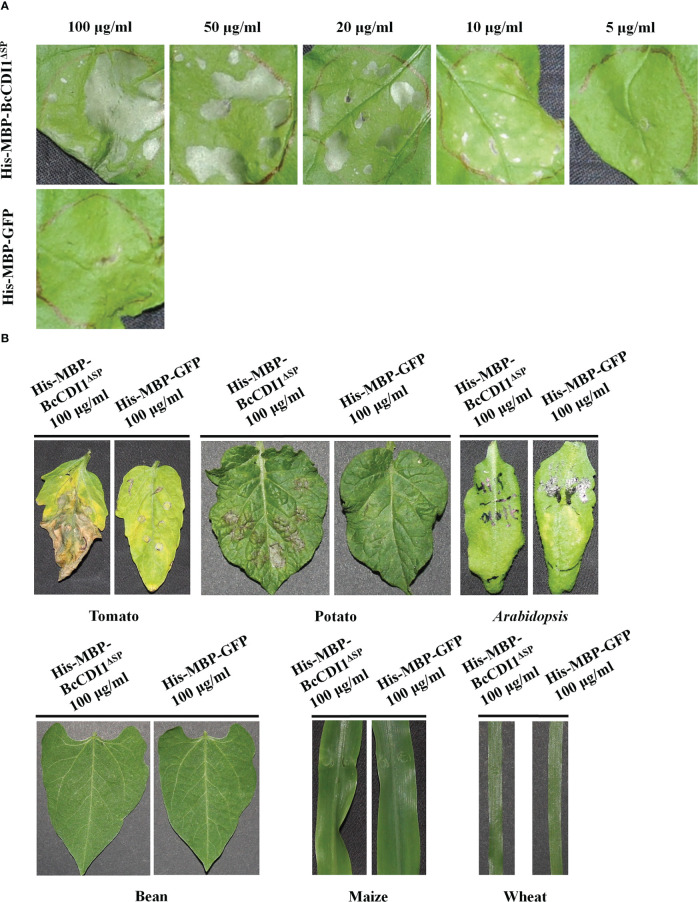
His-MBP-BcCDI1^ΔSP^ protein induces necrosis in tobacco, tomato, and potato. Proteins were produced in *Escherichia coli*, purified, and suspended in PBS. **(A)** Response of *Nicotiana benthamiana* leaves infiltrated with different concentrations of His-MBP-BcCDI1^ΔSP^ at 5 days after treatment. **(B)** Response of tomato, potato, *Arabidopsis*, bean, maize, and wheat leaves infiltrated with 100 μg/ml of protein solution at 5 days after treatment. The His-MBP-GFP protein was used as the control.

To determine whether BcCDI1 can induce necrosis in plants besides *N. benthamiana* and to avoid the *A. tumefaciens* incompatibility effects on plants, we generated the His-MBP-BcCDI1^ΔSP^ protein in *E. coli* (**Supplementary Figure S2**) and infiltrated tobacco leaves with the purified protein at different concentrations using a syringe. As a result, 10 μg/ml of His-MBP-BcCDI1^ΔSP^ recombinant protein was sufficient to trigger leaf necrosis in *N. benthamiana* (**Figure 3A**). The purified His-MBP-BcCDI1^ΔSP^ recombinant protein could also trigger cell death in tomato and potato leaves, but not in bean and *A. thaliana*, or in the monocots wheat and maize (**Figure 3B**).

### *Bccdi1* is induced during infection but is not essential for stress tolerance and pathogenicity

**Figure 4:**
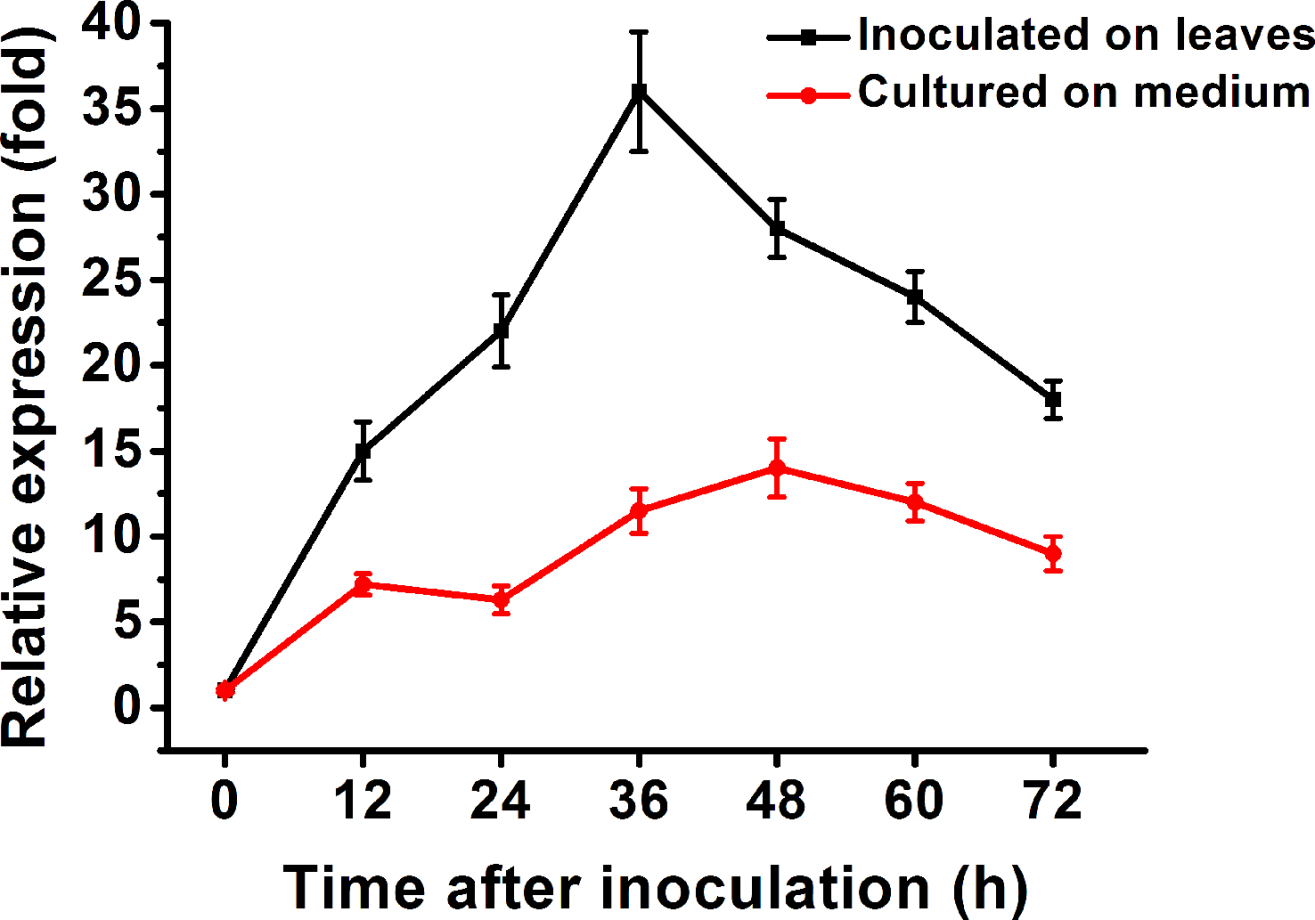
Expression of the *Bccdi1* gene is upregulated during infection. Bean leaves (black line) or Gamborg’s B5 medium (red line) was inoculated with *Botrytis cinerea* conidia, and the expression level of the *Bccdi1* gene was evaluated by RT-qPCR. The expression level of *Bccdi1* inoculated on the plant or in Gamborg’s B5 medium at 0 h was set as 1, and the relative transcript level was calculated using the comparative Ct method. The transcript levels of the *B cinerea bcgpdh* gene were used to normalize different samples. Data represent means and standard deviations of three independent replications.

RT-qPCR was carried out to determine the expression pattern of *Bccdi1*. As a result, the transcript level of *Bccdi1* increased after inoculation on the bean leaves and reached the peak at 36 h post-inoculation (hpi), which was approximately 35-fold that at 0 hpi (**Figure 4**). When *B. cinerea* was inoculated on solid Gamborg’s B5 medium, the transcript level of *Bccdi1* remained stable, and the maximum level was only approximately 12-fold that at 0 hpi (**Figure 4**).

To further clarify the potential role of BcCDI1 in *B. cinerea* infection, transformants with the deletion or overexpression of *Bccdi1* were generated and verified by PCR and RT-qPCR analyses (**Supplementary Figure S3**).

All *Bccdi1* deletion and overexpression transformants exhibited normal conidial production, colony morphology, and growth rate on potato dextrose agar (PDA) as the wild-type strain (**Supplementary Figure S4**). In addition, no statistically significant difference in stress tolerance (0.5 mg/ml of Congo Red, 0.3 mg/ml of Calcofluor White, 1 M of NaCl, 0.02% SDS, and 1 M of sorbitol) was observed between the transformants and wild-type strain (**Supplementary Figure S5**).

**Figure 5:**
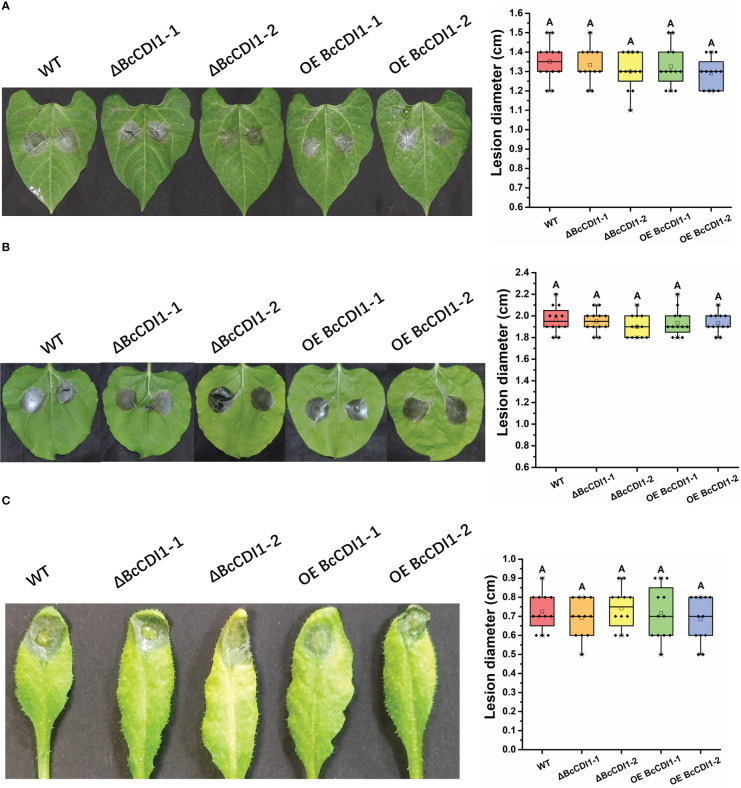
Pathogenicity analysis of *Botrytis cinerea* strain. **(A)** Bean leaves were inoculated with 7.5 μl of conidial suspension (1 × 10^5^ conidia/ml). The plants were incubated in a humid chamber at 22°C for 72 h and photographed, and the lesion size was determined. **(B)** Tobacco leaves were inoculated with 7.5 μl of conidial suspension (1 × 10^5^ conidia/ml). **(C)** *Arabidopsis thaliana* leaves were inoculated with 7.5 μl of conidia suspension (1 × 10^5^ conidia/ml). The plants were incubated in a humid chamber at 22°C for 72 h and photographed, and the lesion size was determined. Data were obtained from three independent experiments, with four replicates in each experiment. In box plots, whiskers indicate the minimum and maximum values, the line indicates the median, and the box boundaries indicate the upper (25th percentile) and lower (75th percentile) quartiles. All data are plotted as black dots. Similar letters in the graph indicate no statistical differences at *P* ≤0.01 using ANOVA (one-way) followed by Tukey’s *post-hoc* test.

The pathogenicity assay revealed that compared with the wild-type strain, the *Bccdi1* deletion or overexpression strains had no significant difference in lesion size on bean, tobacco, and *A. thaliana* leaves (**Figure 5**), indicating that *Bccdi1* has no effect on the outcome of *B. cinerea* infection.

### BcCDI1 induces the resistance of *N. benthamiana* against *B. cinerea*

**Figure 6:**
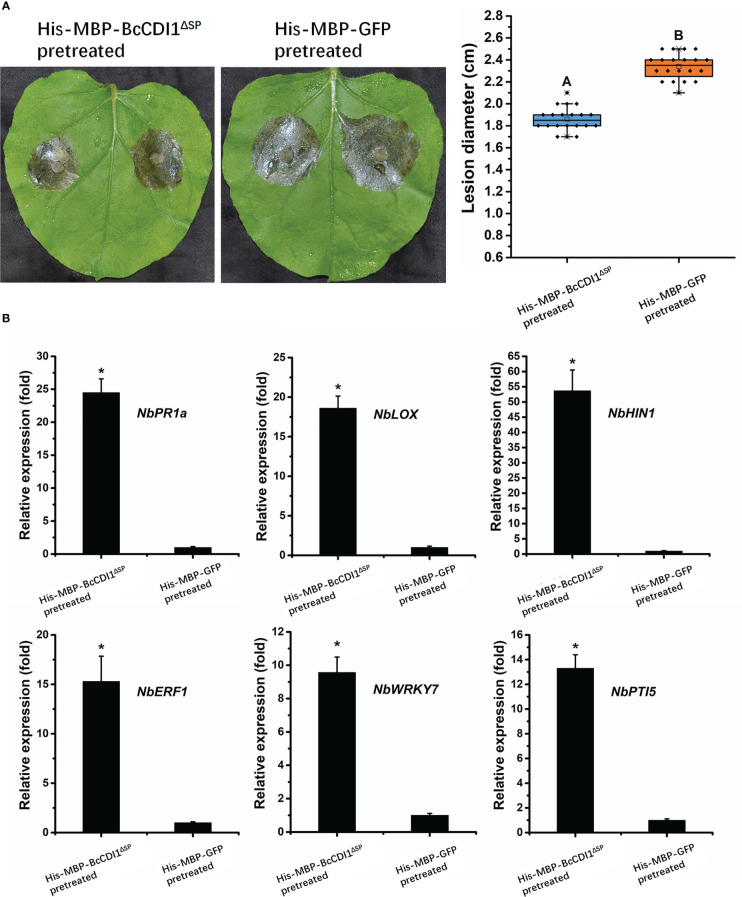
BcCDI1 induces resistance in tobacco. **(A)** *Nicotiana benthamiana* leaves were infiltrated with 5 μg/ml of purified BcCDI1 or GFP protein. After 2 days, the infiltrated leaves were inoculated with *Botrytis cinerea* in a humid chamber. The lesions were photographed and measured at 48 hpi. Data were obtained from three independent experiments with a total of 20 samples. In box plots, whiskers indicate the minimum and maximum values; the line indicates the median; the box boundaries indicate the upper (25th percentile) and lower (75th percentile) quartiles. All data are plotted as black dots. Different letters in the graph indicate statistical differences at *P* ≤0.01 using ANOVA (one-way) followed by Tukey’s *post-hoc* test. **(B)** The relative expression levels of defense-related genes from tobacco leaves treated with BcCDI1 or GFP for 48 h were determined by RT-qPCR analysis. The expression level of the indicated genes in GFP-treated leaves was set as 1. The expression level of the tobacco *NbEF1α* gene was used to normalize different samples. Data represent means and standard deviations of three independent replicates. Asterisks in the graph indicate statistical differences at *P* ≤0.01 using ANOVA (one-way) followed by Tukey’s *post-hoc* test.

Recently, several studies have revealed that some necrosis-inducing proteins can be recognized by the plant immune system and activates defense response. To determine whether BcCDI1 can trigger plant resistance, 5 μg/ml of purified His-MBP-BcCDI1^ΔSP^ protein or His-MBP-GFP was injected into *N. benthamiana* leaves using a syringe. After 48 h, the infiltrated leaves were inoculated with *B. cinerea* and incubated in a chamber for an additional 48 h. The results showed that tobacco leaves pretreated with His-MBP-BcCDI1^ΔSP^ had an obviously smaller lesion size than those pretreated with His-MBP-GFP protein (**Figure 6A**), suggesting that the BcCDI1 protein can induce plant resistance to *B. cinerea* infection.

To test whether the promoting effect of His-MBP-BcCDI1^ΔSP^ on the resistance of tobacco is associated with changes in the expression of defense-related genes, RT-qPCR was performed to analyze the changes in the transcripts of the salicylic acid (SA) signal pathway gene *NbPR1a*, the jasmonic acid (JA) signal pathway gene *NbLOX*, the HR-related gene *HIN1*, the ethylene signal pathway gene *NbERF1*, and the PTI-related genes *NbWRKY7* and *NbPTI5* as previously described (Zhu et al., 2022). The results demonstrated that the transcripts of these defense-related genes in *N. benthamiana* leaves dramatically increased after infiltration of the His-MBP-BcCDI1^ΔSP^ protein for 24 h compared with those in His-MBP-GFP-infiltrated leaves (**Figure 6B**). A similar result was reported in a previous study, in which the homolog of BcCDI1 in *Rhynchosporium commune* could also induce the transcriptional upregulation of defense-related genes in *N. benthamiana* leaves (Franco-Orozco et al., 2017).

We also determined whether BcCDI1 can trigger systemic resistance in non-treated leaves of the same tobacco plants. The leaves were infiltrated with 100 μg/ml of purified His-MBP-BcCDI1^ΔSP^ or His-MBP-GFP protein. After 2 days, the untreated leaves of the same plant were inoculated with *B. cinerea*, and the plant was incubated in a humid chamber for an additional 48 h. As a result, the His-MBP-BcCDI1^ΔSP^ pretreated plants showed no obvious difference in disease lesion from the His-MBP-GFP pretreated plants (**Supplementary Figure S6**), indicating that His-MBP-BcCDI1^ΔSP^ cannot induce plant systemic resistance.

### BAK1 and SOBIR1 are required for the necrosis-inducing activity of BcCDI1 in *Nicotiana benthamiana*

**Figure 7:**
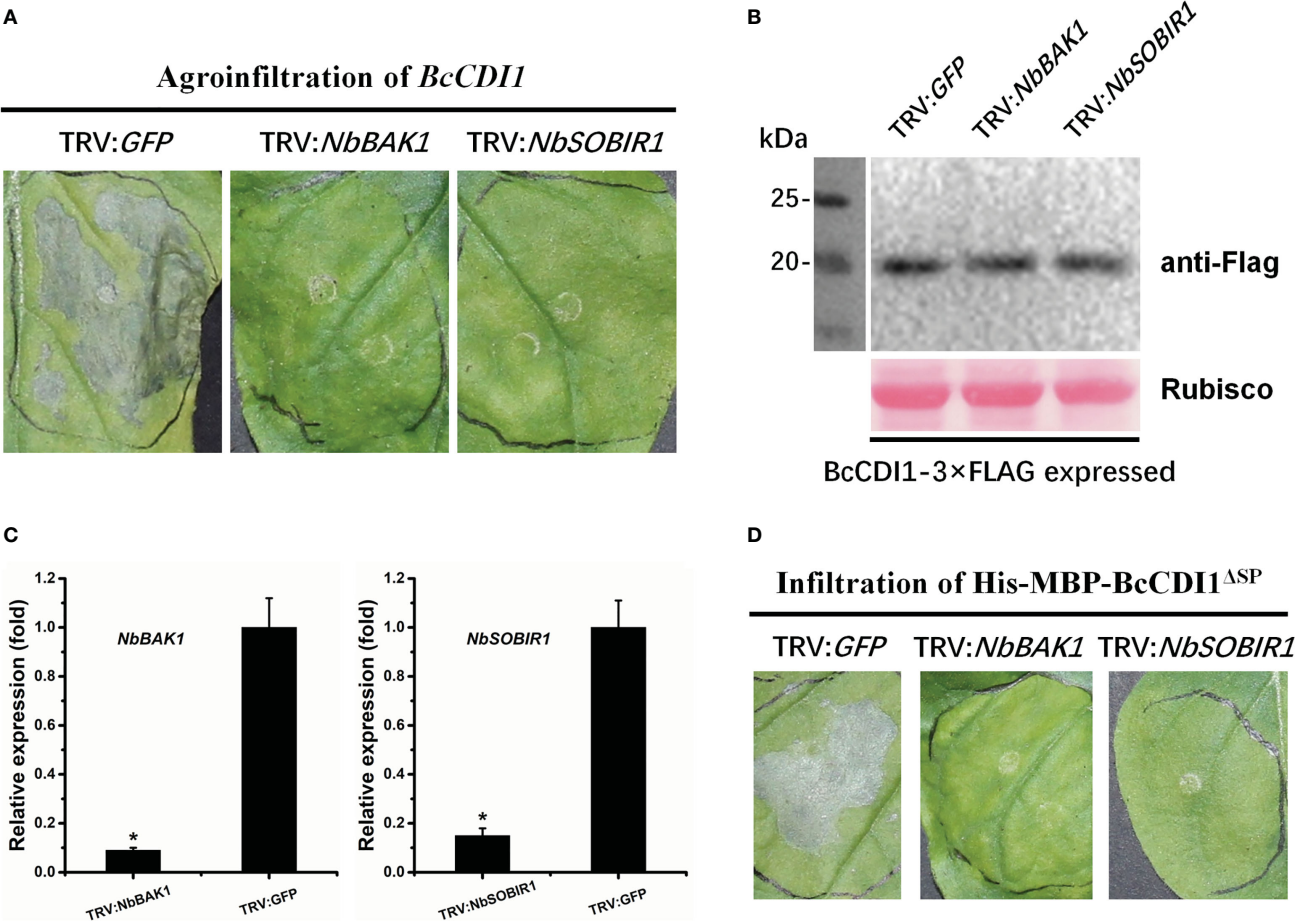
The necrosis-inducing activity of BcCDI1 is mediated by *NbBAK1* and *NbSOBIR1*. **(A)** Three weeks after the initiation of VIGS, *BcCDI1-3×Flag* was transiently expressed in the gene-silenced leaves using agroinfiltration. The leaves were photographed 5 days after treatment. **(B)** Immunoblot analysis of proteins from the indicated *Nicotiana benthamiana* leaves with transient expression of *BcCDI1-3×Flag*. Top panel: BcCDI1-3×Flag was detected using the anti-Flag antibody; bottom panel, staining of the Rubisco large subunit with Ponceau S. **(C)** *NbBAK1* and *NbSOBIR1* expression levels in gene-silenced tobacco leaves were determined by RT-qPCR analysis. The expression level in the control plants (TRV: *GFP*) was set as 1. *NbEF1α* was used as an endogenous control. Data represent means and standard deviations from three biological replicates. Asterisks indicate significant differences (*P* ≤ 0.01) followed by Tukey’s *post-hoc* test. **(D)** Response of gene-silenced leaves infiltrated with 100 μg/ml of protein solution at 5 days after treatment.

Since BcCDI1 is a secreted effector localized in the apoplastic space, it may interact with plant membrane RLKs or receptor-like proteins (RLPs) to transduce the immunity signals via the RLP–SOBIR1–BAK1 complex into the intracellular space, which is similar to the function of other apoplast-localized effector proteins (Liebrand et al., 2013; Zhang et al., 2014; Albert et al., 2015; Ma et al., 2015; Postma et al., 2016; Gui et al., 2017; Zhu et al., 2017). Therefore, we generated *NbBAK1*- and *NbSOBIR1*-silenced *N. benthamiana* using VIGS, which were then agroinfiltrated with *Agrobacterium* harboring BcCDI1 expression construct. The results showed that BcCDI1 failed to cause necrosis in *BAK1*- or *SOBIR1*-silenced tobacco leaves, whereas the control plant leaves infiltrated with pTRV2-*GFP* showed severe cell death after agroinfiltration of *BcCDI1* or infiltration of purified His-MBP-BcCDI1^ΔSP^ protein (**Figure 7**), indicating that both BAK1 and SOBIR1 can mediate the necrosis-inducing activity of BcCDI1, possibly through an unknown RLK or RLP.

## Discussion

Plant pathogens can secrete effectors to promote host colonization through diverse molecular mechanisms. In *B. cinerea*, many predicted secreted proteins have been mined through comparative transcriptomics, secreted proteomics, or genomics approaches (Zhu et al., 2017; Mousavi-Derazmahalleh et al., 2019; Valero-Jiménez et al., 2019; Denton-Giles et al., 2020; Zhang et al., 2020; Jeblick et al., 2023). However, due to the high functional redundancy of virulence factors, only a limited number of secreted proteins have been confirmed to have a true contribution to the pathogenesis of *B. cinerea* (Brito et al., 2006; Frías et al., 2011; Frías et al., 2016; Zhu et al., 2017; Bi et al., 2022; Leisen et al., 2022). In the present study, putative secreted proteins predicted from *B. cinerea* genomics data were screened by transient expression using the agroinfiltration assay. A secreted protein, BcCDI1, was identified as a candidate effector protein involved in the invasion of *B. cinerea*, which could simultaneously cause necrosis and induce host resistance in tobacco.

Expression analysis revealed that the expression of *Bccdi1* was upregulated at the early infection stage (0-36 hpi) of *B. cinerea*, reaching the peak at 36 hpi and declining thereafter. It has been demonstrated that the successful establishment of pathogen infection largely depends on the killing of a sufficient number of host cells to create a region of dead plant tissue, in which fungal biomass will be accumulated before the transition to the intermediate and then late infection phases (Shlezinger et al., 2011; Eizner et al., 2017). The specific activation of *Bccdi1 in planta* and induction of transcription at the early infection stage (**Figure 4**) indicate that BcCDI1 belongs to the array of CDIPs in *B. cinerea.* However, despite the evidently important role of BcCDI1 in *B. cinerea* pathogenicity, the deletion or overexpression transformants showed no significant difference in lesion size from the wild-type strain (**Figure 5**), which is not surprising considering the high functional redundancy of virulence factors in the fungal secretome (Zhu et al., 2017; Leisen et al., 2022). Indeed, similar conclusions have been reported in the analysis of other *B. cinerea* CIDPs, such as BcNEP1/2 (Arenas et al., 2010), BcIEB1 (Frías et al., 2016), BcXYG1 (Zhu et al., 2017), and BcCrh1 (Bi et al., 2021). Previous studies with the assistance of the PathTrack^©^ system (Eizner et al., 2017) discovered earlier and more intense local necrosis caused by BcXYG1 overexpression strains. However, the lesion size showed no notable difference from that of the wild-type strain at the later infection stage, which can explain the similar lesion size at 72 hpi (Zhu et al., 2017). Hence, a more detailed analysis of pathogenicity, such as the use of PathTrack^©^, will better reveal the possible role of BcCDI1 at the early infection stage.

Recently, there has been increasing evidence suggesting that the defense stimulation of these CDIPs is not related to the necrosis-inducing activity (Oome et al., 2014; Zhu et al., 2017; Bi et al., 2021), suggesting that different plant receptors may be involved in host immune response and necrosis-inducing pathways. Necrotrophic fungi cause significant crop loss worldwide. Hence, it may be feasible to improve engineering crops with broad and durable disease resistance without damaging the host cells based on mutant CDIPs from *B. cinerea* that have no necrosis-inducing activity. Further research is needed to determine the relationship between necrosis-inducing activity and host defense-triggering activity.

Effector repertoire can be defined into two categories: the effectors either remain at the plant–pathogen interface (termed apoplastic effectors) or are taken up by host cells (translocated or cytoplasmic effectors) (Asai and Shirasu, 2015). To date, over 15 CDIPs have been characterized in *B. cinerea*. Most of them remain in the apoplastic space after secretion by the fungus, except for the transglycosylase BcCrh1, which causes plant cell death depending on cytoplasmic localization (Bi et al., 2022). The BcCDI1 protein reported here belongs to the apoplast-localized CDIPs (**Figure 2**), whose cell death-inducing activity is mediated by plant extracellular membrane components. Plants employ RLKs and RLPs, which are categorized according to the presence or absence of a cytoplasmic kinase domain, as PRRs to monitor their apoplastic environment and sense non-self and damaged-self patterns as signs of potential danger. BAK1 and SOBIR1 have been described as the most widespread co-receptors during the last decade of inward signaling through RLKs and RLPs (Boutrot and Zipfel, 2017). Our results demonstrated that BcCDI1 is an apoplast-localized CDIP, and its necrosis-inducing activity is dependent on NbBAK1/NbSOBIR1 and possibly mediated by an unknown RLK or RLP. Similarly, it has been reported that the necrosis-inducing activity of the BcCDI1 homolog in *R. commune* is also dependent on NbBAK1 and NbSOBIR1 (Franco-Orozco et al., 2017). Further studies can be conducted to improve our understanding of the molecular details involved in the BAK1- and SOBIR1-dependent recognition of BcCDI1.

To develop new strategies for controlling plant diseases caused by pathogenic fungi, it is important to fully understand the pathogenic mechanisms of fungi, the evolution of virulence traits, the molecular basis for host adaptation, and particularly how to engineer and improve the crop resistance in a more sustainable way (Doehlemann et al., 2017). The panoramic view of the invasion model and virulence factors of *B. cinerea* has been intensively studied. Although the specific molecular mechanisms underlying *B. cinerea* invasion remain elusive, it is becoming increasingly evident that the infection process of *B. cinerea* is far more sophisticated and complex than previously estimated.

In conclusion, exploration of the underlying pathogenic mechanisms of BcCDI1, an effector from *B. cinerea*, paves the way for the development of novel strategies to control this devastating disease.

## Conclusions

This study characterized the secreted protein BcCDI1 from *B. cinerea*. Our results demonstrate that the *BcCDI1* gene does not affect the outcome of *B. cinerea* infection or other phenotypes including conidial production, colony morphology, growth rate, and stress tolerance. Further analysis demonstrated that BcCDI1 can induce plant cell death by interacting with an unknown RLP in the apoplastic space of plant cells to transduce the immunity signals via the RLP–SOBIR1–BAK1 complex into the intracellular space and trigger plant immune response. Our findings suggest that BcCDI1 has the potential to be applied in breeding crops with resistance to gray mold and other destructive pathogenic microbes.

## Data availability statement

The datasets presented in this study can be found in online repositories. The names of the repository/repositories and accession number(s) can be found in the article/**Supplementary Material**.

## Author contributions

Conceived and designed the experiments: WZ, HD, and KB. Performed the experiments: WZ, HD, RX, and JY. Analyzed the experiment data: WZ, HD, CX, D-zY, JW, RX, and JY. Contributed reagents/materials/analysis tools: WZ, RX, JY, and KB. Wrote the paper: WZ, RX, JY, and KB. All authors contributed to the article and approved the submitted version.

## Supplementary material

Supplementary Figure 1 Sequence similarities between BcCDI1 and its homologues. **(A)** Multiple sequence alignment of BcCDI1 and its homologues. Full-length protein sequences were aligned using Clustal W and the alignment was edited using Jalview. Intensity of blue shading reflects the level of amino acid identity at each position. Bc: *B. cinerea* BcCDI1 (BCIN06g00550); Zt: *Zymoseptoria tritici* (XP_003847964.1, E-value: 5.58e-016, 48.8% identity); Uv: *Ustilaginoidea virens* (KDB13604.1, E-value: 2.27e-034, 55.2% identity); Rc: *Rhynchosporium commune* (KX499545, E-value: 2.93e-039, 45.2% identity); Ss: *Sclerotinia sclerotiorum* (XP_001589511.1, E-value: 2.41e-049, 74.7% identity); Pn: *Parastagonospora nodorum* (XP_001792149.1, E-value: 2.78e-021, 43.5% identity); Bg: *Blumeria graminis f.* sp. *hordei* (CCU82201.1, E-value: 2.25e-021, 39% identity); Cg: *Colletotrichum graminicola* (CAQ16238.1, E-value: 2.49e-019, 40.4% identity). **(B)** Phylogenetic analysis of BcCDI1 and its homologues from other fungi. The full-length protein sequences were analyzed using MEGA X with Unrooted neighbor-joining bootstrap (1000 replicates). The black circle marks the location of BcCDI1. A scale bar at the lower left corresponds to a genetic distance of 0.2.

Supplementary Figure 2 Expression of recombinant proteins. SDS-PAGE analysis of purified His-MBP-BcCDI1^ΔSP^ and His-MBP-GFP proteins from *E. coli* stained with Coomassie Blue.

Supplementary Figure 3 Deletion and verification of the *Bccdi1* gene in *B. cinerea*. **(A)** Strategy used to generate the *Bccdi1* gene deletion mutants. The deletion construct used to transform the wild-type strain contained the hygromycin resistance (*hph*) cassette flanked by upstream and downstream sequence of the *Bccdi1* gene. The positions of the PCR primers used to verify the deletion transformants are indicated (*Bccdi1* Del-up F and *Bccdi1* Del-down R). The scale bar indicates 500 bp. **(B)** PCR amplification to verify the *Bccdi1* deletion mutants using the 5’flank For and 3’flank Rev primers. As templates, genomic DNA from either the wild-type strain or the *Bccdi1* deletion mutants ΔBcCDI1-1 and ΔBcCDI1-2 was used as indicated. **(C)** RT-qPCR was carried out to analyze the expression levels of the *Bccdi1* gene in the wild-type strain, the *Bccdi1* deletion mutants ΔBcCDI1-1 and ΔBcCDI1-2, and the *Bccdi1* overexpression strains OE BcCDI1-1 and OE BcCDI1-2. The relative transcript levels were calculated using the comparative Ct method. The *Bccdi1* gene expression level in the wild-type strain was set as 1. The transcript level of the *B. cinerea bcgpdh* gene was used to normalize different samples. Data represent means and standard deviations of three independent replicates. ND = not detected.

Supplementary Figure 4 Phenotypes of wild-type, and *Bccdi1* deletion and overexpression strains of *B. cinerea*. **(A)** Colony morphology, sporulation, and sclerotia formation. Top: colonies on PDA at 22°C for 15 d in complete darkness. Bottom: *B. cinerea* strains on PDA plates at 22°C for 7 d with continuous fluorescent light. **(B)** Hyphal growth rate. *B. cinerea* strains were grown on PDA plates at 22°C with continuous fluorescent light. Radial growth was measured every day for 4 d and the growth rate was calculated. Data represent means and standard deviations of three independent replicates. Same letters in the graph indicate no statistical difference at P ≤ 0.01 using one-way ANOVA followed by Tukey’s *post hoc* test. **(C)** Conidial production of indicated strains cultured on PDA plates at 22°C for 7 d. Conidiation of each strain was determined by collecting and counting conidia with a hematocytometer. Data represent means and standard deviations of three independent replicates. Same letters in the graph indicate no statistical difference at P ≤ 0.01 using one-way ANOVA followed by Tukey’s *post hoc* test. Same letters in the graph indicate no statistical difference at P ≤ 0.01 using one-way ANOVA followed by Tukey’s *post hoc* test.

Supplementary Figure 5
*Bccdi1* deletion does not affect stress tolerance of *B. cinerea*. Inhibition rate of the radial growth of the wild-type strain, *Bccdi1* deletion mutants and *Bccdi1* overexpression strains was analyzed on PDA plates supplemented with 0.5 mg/ml Congo Red, 0.3 mg/ml Calcofluor White, 1 M NaCl, 0.02% SDS and 1 M sorbitol at 22°C, respectively. Data represent means and standard deviations from three independent experiments, each with three replications. Same letters in the graph indicate no statistical differences at P ≤ 0.01 using ANOVA (one-way) followed by Tukey’s *post hoc* test.

Supplementary Figure 6 BcCDI1 does not induce systemic resistance in tobacco. *N. benthamiana* leaves were infiltrated with 100 μg/ml purified His-MBP-BcCDI1^ΔSP^ or His-MBP-GFP protein. After 2 d, the non-treated leaves of the same plant were inoculated with *B. cinerea* in a humid chamber. The lesions were photographed and measured at 48 hpi. All data were obtained from three independent experiments with a total of 12 samples. In box plots, whiskers indicate the minimum and maximum values; the line indicates the median; the box boundaries indicate the upper (25th percentile) and lower (75th percentile) quartiles. All data are plotted as black dots. The same letters in the graph indicate no statistical difference at P ≤ 0.01 using ANOVA (one-way) followed by Tukey´s *post hoc* test.
