# Supplementary figures and images for: Botrytis cinerea BcCDI1 protein triggers both plant cell death and immune response
Source: Front Plant Sci. 2023 Apr 25;14:1136463. doi: 10.3389/fpls.2023.1136463 (PMC10167277; doi:10.3389/fpls.2023.1136463)

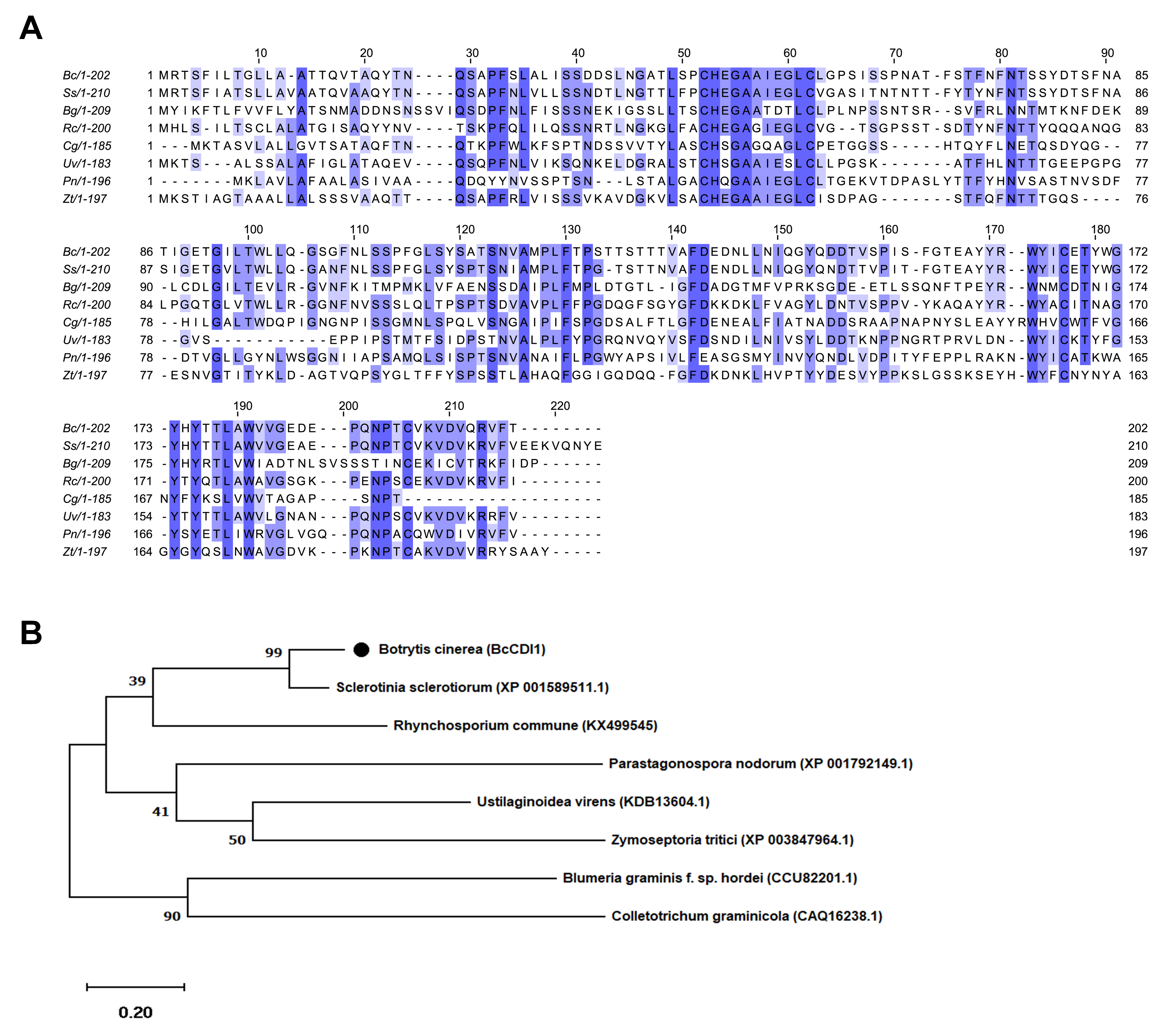

Supplement: Supplementary Figure 1 — Sequence similarities between BcCDI1 and its homologues. (A) Multiple sequence alignment of BcCDI1 and its homologues. Full-length protein sequences were aligned using Clustal W and the alignment was edited using Jalview. Intensity of blue shading reflects the level of amino acid identity at each position. Bc: B. cinerea BcCDI1 (BCIN06g00550); Zt: Zymoseptoria tritici (XP_003847964.1, E-value: 5.58e-016, 48.8% identity); Uv: Ustilaginoidea virens (KDB13604.1, E-value: 2.27e-034, 55.2% identity); Rc: Rhynchosporium commune (KX499545, E-value: 2.93e-039, 45.2% identity); Ss: Sclerotinia sclerotiorum (XP_001589511.1, E-value: 2.41e-049, 74.7% identity); Pn: Parastagonospora nodorum (XP_001792149.1, E-value: 2.78e-021, 43.5% identity); Bg: Blumeria graminis f. sp. hordei (CCU82201.1, E-value: 2.25e-021, 39% identity); Cg: Colletotrichum graminicola (CAQ16238.1, E-value: 2.49e-019, 40.4% identity). (B) Phylogenetic analysis of BcCDI1 and its homologues from other fungi. The full-length protein sequences were analyzed using MEGA X with Unrooted neighbor-joining bootstrap (1000 replicates). The black circle marks the location of BcCDI1. A scale bar at the lower left corresponds to a genetic distance of 0.2. [file Image_1.tif]

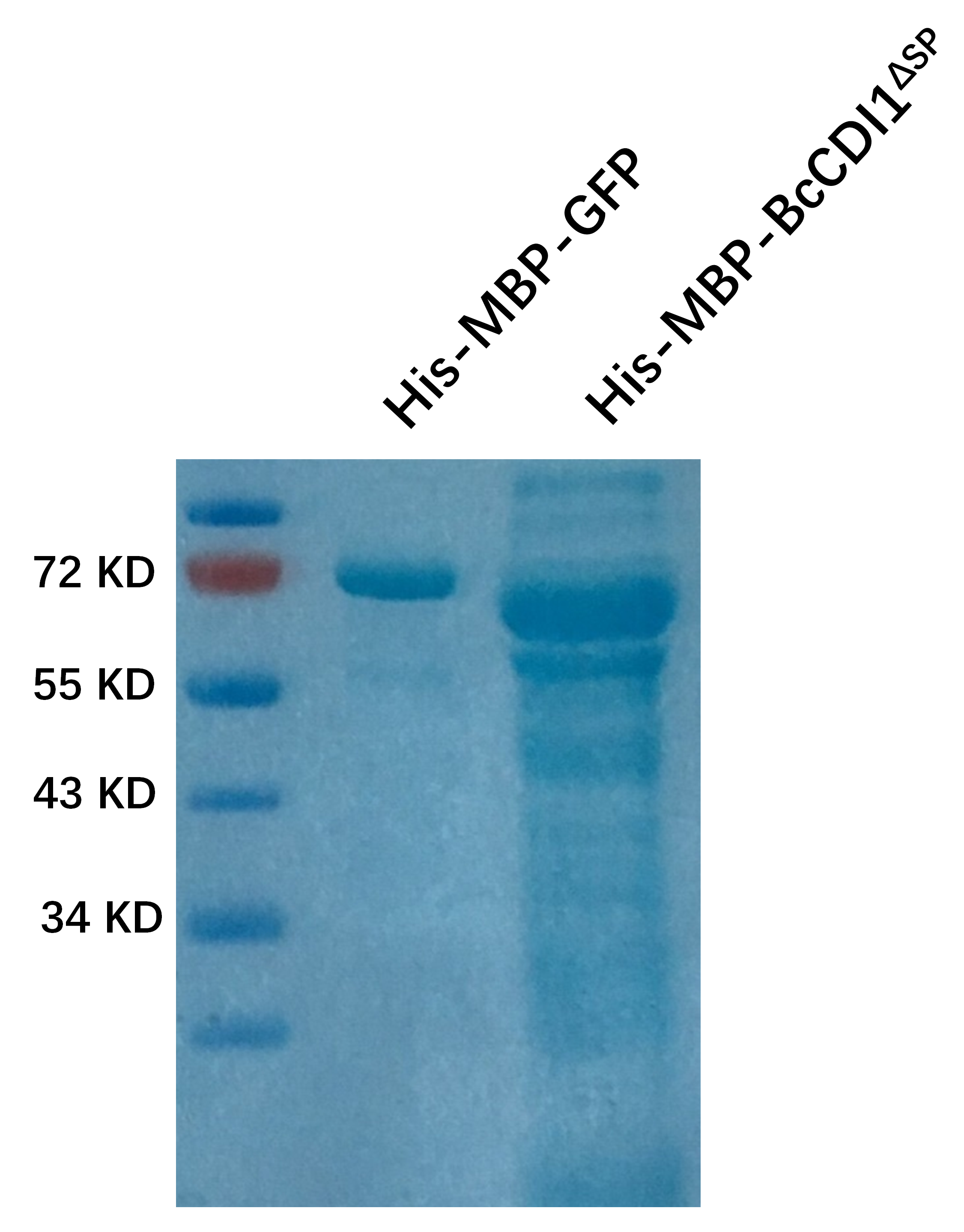

Supplement: Supplementary Figure 2 — Expression of recombinant proteins. SDS-PAGE analysis of purified His-MBP-BcCDI1ΔSP and His-MBP-GFP proteins from E. coli stained with Coomassie Blue. [file Image_2.tif]

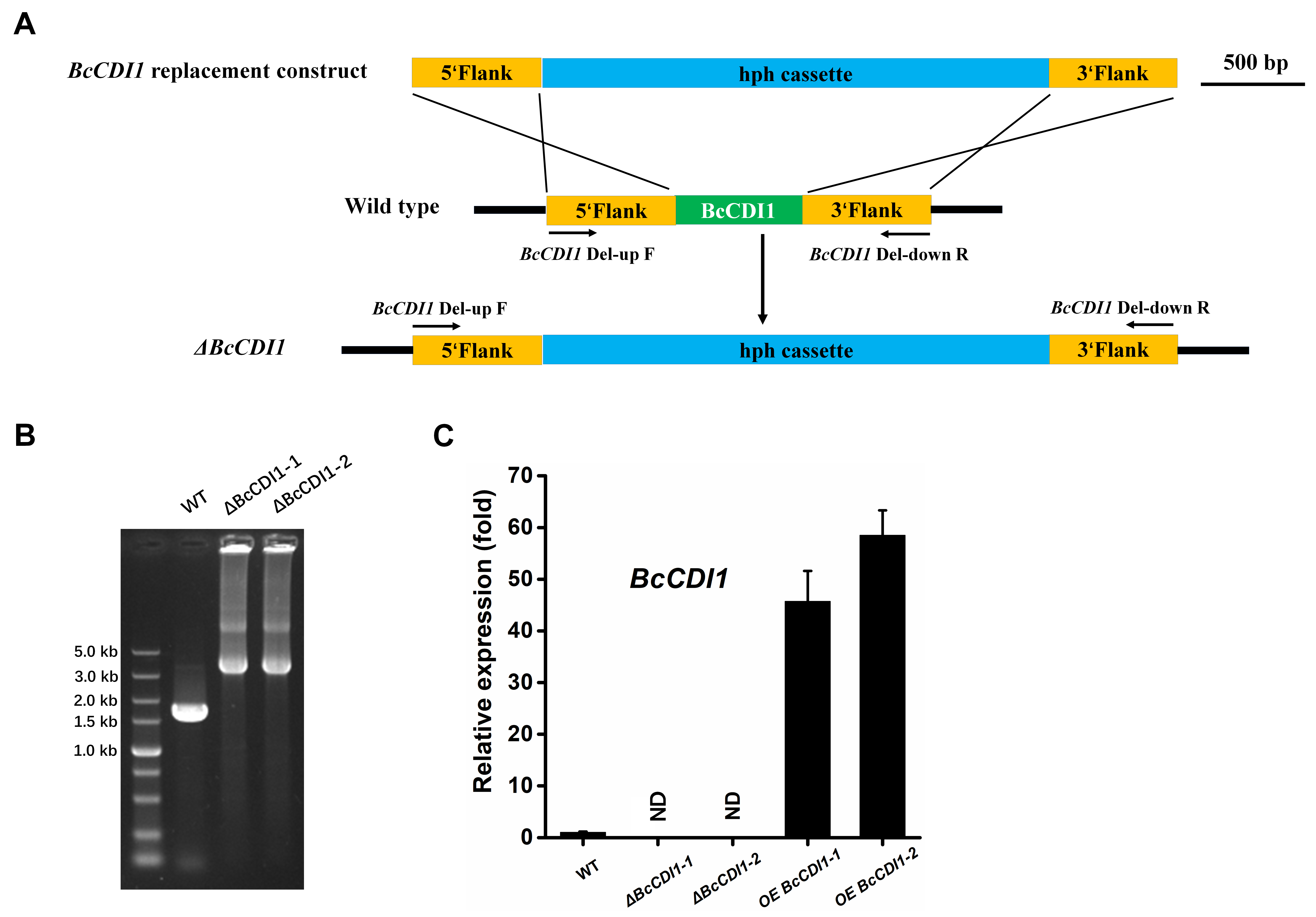

Supplement: Supplementary Figure 3 — Deletion and verification of the Bccdi1 gene in B. cinerea. (A) Strategy used to generate the Bccdi1 gene deletion mutants. The deletion construct used to transform the wild-type strain contained the hygromycin resistance (hph) cassette flanked by upstream and downstream sequence of the Bccdi1 gene. The positions of the PCR primers used to verify the deletion transformants are indicated (Bccdi1 Del-up F and Bccdi1 Del-down R). The scale bar indicates 500 bp. (B) PCR amplification to verify the Bccdi1 deletion mutants using the 5’flank For and 3’flank Rev primers. As templates, genomic DNA from either the wild-type strain or the Bccdi1 deletion mutants ΔBcCDI1-1 and ΔBcCDI1-2 was used as indicated. (C) RT-qPCR was carried out to analyze the expression levels of the Bccdi1 gene in the wild-type strain, the Bccdi1 deletion mutants ΔBcCDI1-1 and ΔBcCDI1-2, and the Bccdi1 overexpression strains OE BcCDI1-1 and OE BcCDI1-2. The relative transcript levels were calculated using the comparative Ct method. The Bccdi1 gene expression level in the wild-type strain was set as 1. The transcript level of the B. cinerea bcgpdh gene was used to normalize different samples. Data represent means and standard deviations of three independent replicates. ND = not detected. [file Image_3.tif]

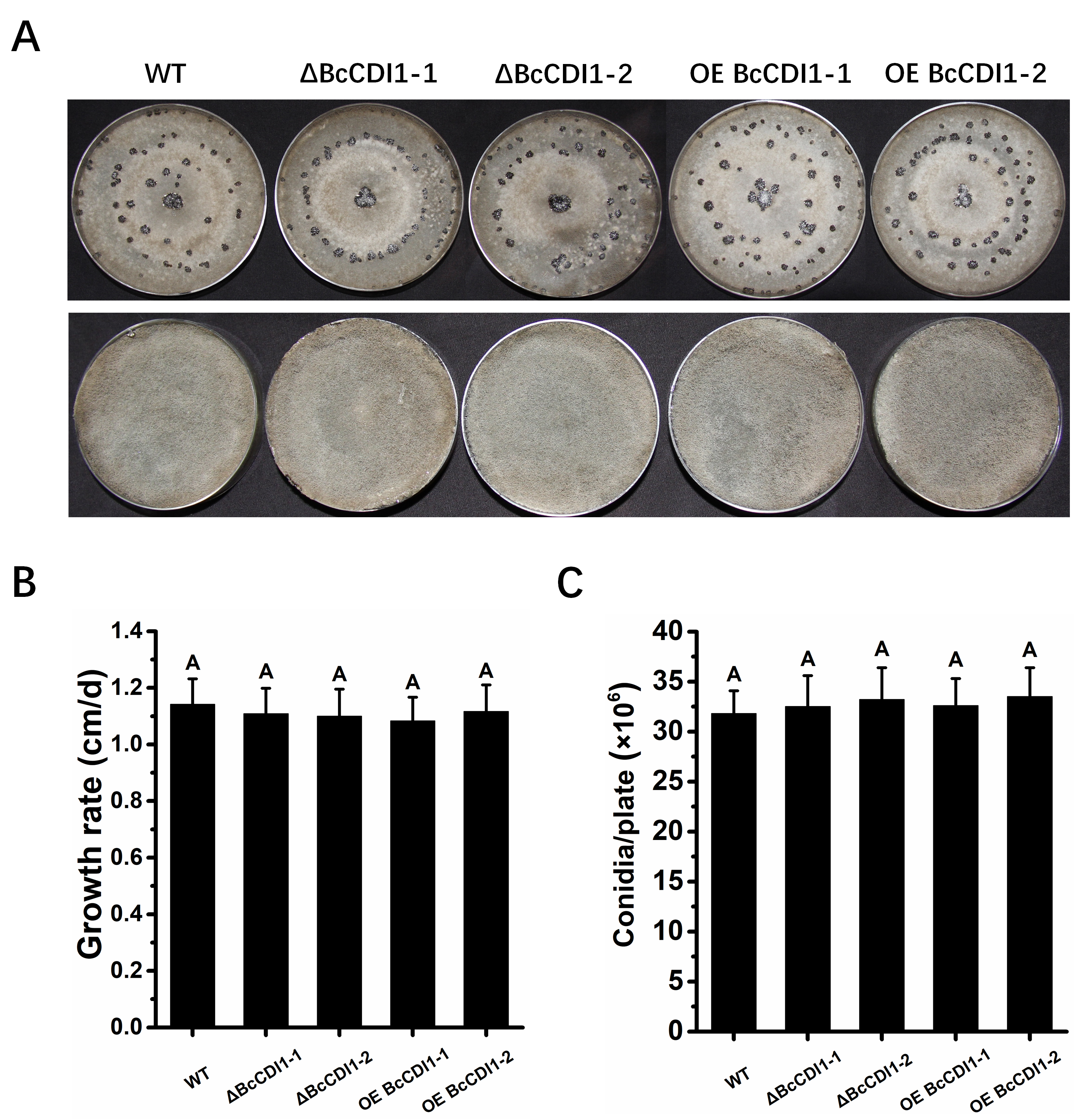

Supplement: Supplementary Figure 4 — Phenotypes of wild-type, and Bccdi1 deletion and overexpression strains of B. cinerea. (A) Colony morphology, sporulation, and sclerotia formation. Top: colonies on PDA at 22°C for 15 d in complete darkness. Bottom: B. cinerea strains on PDA plates at 22°C for 7 d with continuous fluorescent light. (B) Hyphal growth rate. B. cinerea strains were grown on PDA plates at 22°C with continuous fluorescent light. Radial growth was measured every day for 4 d and the growth rate was calculated. Data represent means and standard deviations of three independent replicates. Same letters in the graph indicate no statistical difference at P ≤ 0.01 using one-way ANOVA followed by Tukey’s post hoc test. (C) Conidial production of indicated strains cultured on PDA plates at 22°C for 7 d. Conidiation of each strain was determined by collecting and counting conidia with a hematocytometer. Data represent means and standard deviations of three independent replicates. Same letters in the graph indicate no statistical difference at P ≤ 0.01 using one-way ANOVA followed by Tukey’s post hoc test. Same letters in the graph indicate no statistical difference at P ≤ 0.01 using one-way ANOVA followed by Tukey’s post hoc test. [file Image_4.tif]

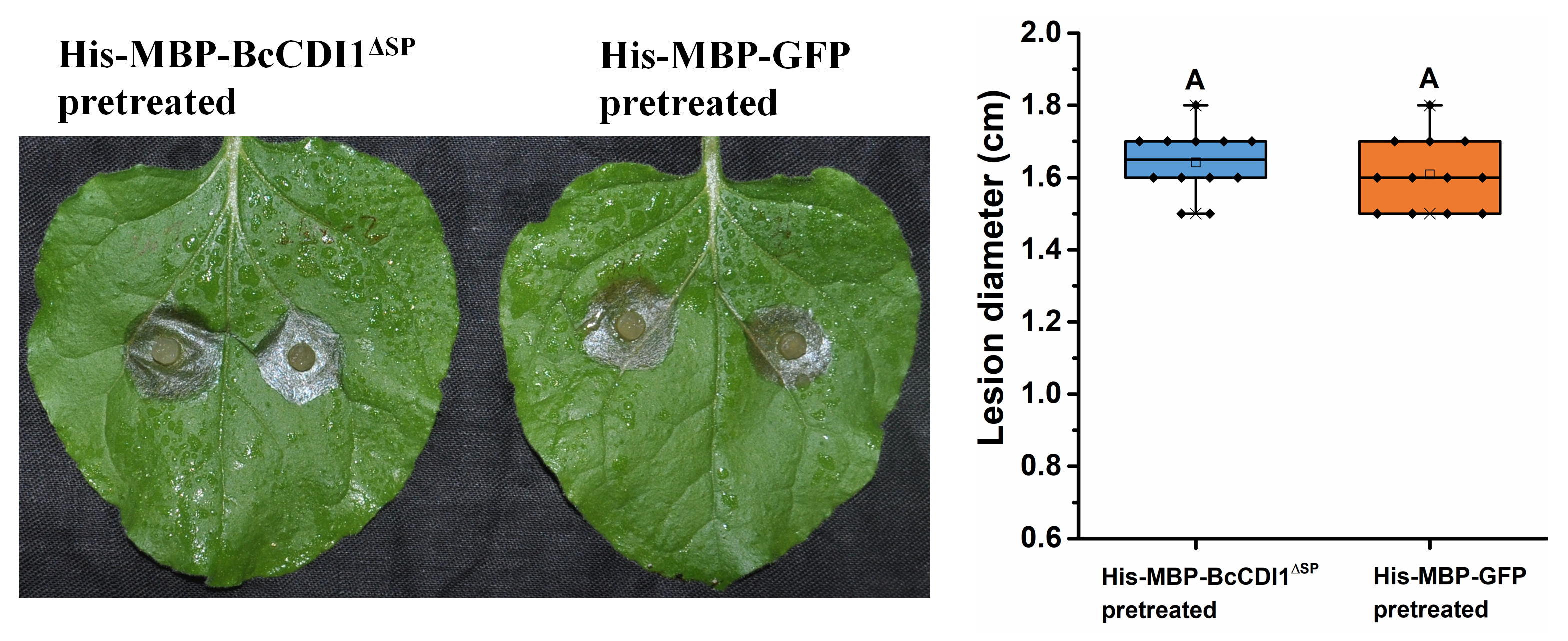

Supplement: Supplementary Figure 6 — BcCDI1 does not induce systemic resistance in tobacco. N. benthamiana leaves were infiltrated with 100 μg/ml purified His-MBP-BcCDI1ΔSP or His-MBP-GFP protein. After 2 d, the non-treated leaves of the same plant were inoculated with B. cinerea in a humid chamber. The lesions were photographed and measured at 48 hpi. All data were obtained from three independent experiments with a total of 12 samples. In box plots, whiskers indicate the minimum and maximum values; the line indicates the median; the box boundaries indicate the upper (25th percentile) and lower (75th percentile) quartiles. All data are plotted as black dots. The same letters in the graph indicate no statistical difference at P ≤ 0.01 using ANOVA (one-way) followed by Tukey´s post hoc test. [file Image_6.tif]
