# Supplementary material for: Botrytis cinerea BcCDI1 protein triggers both plant cell death and immune response
Source: Front Plant Sci. 2023 Apr 25;14:1136463. doi: 10.3389/fpls.2023.1136463 (PMC10167277; doi:10.3389/fpls.2023.1136463)
Supplement: Supplementary file 7 [file Table_1.docx]

**SUPPLEMENTARY TABLE S1. Primers used for vector construction and PCR.**

| **Primer purpose Primer name and sequence** | |
| --- | --- |
| *Bccdi1* deletion for upstream | *Bccdi1* Del-up F: 5’GGTATTGTATGCGGGGTAGTCAAC 3’  *Bccdi1* Del-up R: 5’CACAAAGTCGAATATCAGAGTTTAACACTGTGAGAATAATTCCTGAAAAAAAAAGG 3’ |
| *Bccdi1* deletion for downstream | *Bccdi1* Del-down F: 5’GATCTAGATGCATTCGCGAGGTACCGAGCTACCGAGGTTTGTTAATAGTTGGTAG 3’  *Bccdi1* Del-down R: 5’ TGCGTCCAGCCTCCATTGCCTACA 3’ |
| *Bccdi1* over expression | *Bccdi1-*OE F: 5’ GGCGCGCCATGCGTACCTCATTTATCCTCACTG 3’  *Bccdi1-*OE R: 5’ GCGGCCGCCTAGGTGAAAACTCTTTGAACATC 3’ |
| *Bccdi1* transient expression | *Bccdi1*-TE R: 5’ TCTAGAATGCGTACCTCATTTATCCTCACTG 3’  *Bccdi1*-TE R: 5’ CCCGGGGGTGAAAACTCTTTGAACATC 3’ |
| *Bccdi1^ΔSP^* transient expression | *Bccdi1^ΔSP^*-TE R: 5’ TCTAGAATGCAATACACAAACCAATCCGCTCCAT 3’  *Bccdi1^ΔSP^*-TE R: 5’ CCCGGGGGTGAAAACTCTTTGAACATC 3’ |
| *hph* cassette | *hph* cassette F: 5’ AGCTCGGTACCTCGCGAATGCATCTAGATC 3’  *hph* cassette R: 5’ ACTGTGAGAATAATTCCTGAAAAAAAAAGG 3’ |
| *NAT* cassette | *NAT* cassette F: 5’ GAGCCGCATTCCCGATTC 3’  *NAT* cassette R: 5’ ACTGTGAGAATAATTCCTGAAAAAAAAAGG 3’ |
| *Bccdi1* for RT-qPCR | *Bccdi1*-q F: 5’ CGATAGCCTGAACGGTGCCACTCTT 3’  *Bccdi1*-q R: 5’ TGTCGTAGGAGGAAGTGTTGAAGTT 3’ |
| *Bcgpdh* for RT-qPCR | *Bcgpdh*-q F: 5’CGAAGAATAGCACAAACAGCTGGAC 3’  *Bcgpdh*-q R: 5’CGTCACCTTATGCTTCTTGCTCC 3’ |
| *GFP* expression in *E. coli* | *GFP* His-MBP F: 5’ CATATGGTGAGCAAGGGCGAGGAG 3*’*  *GFP* His-MBP R: 5’ CTCGAGTTACTTGTACAGCTCGTCCATGCCG 3’ |
| *Bccdi1* expression in *E. coli* | *Bccdi1* His-MBP F: 5’ CAATACACAAACCAATCCGCTCC 3’  *Bccdi1* His-MBP R: 5’ TTAGGTGAAAACTCTTTGAACATCAAC 3’ |
| *NbBAK1* for RT-qPCR | *NbBAK1*-q F: 5’GAGGTGGGAGGAATGGCAAA 3’  *NbBAK1*-q R: 5’TTGGCCCCGACAATTCATCT 3’ |
| *NbSOBIR1* for RT-qPCR | *NbSOBIR1*-q F: 5’CCAGCAAGTCACAGAAGGGA 3’  *NbSOBIR1*-q R: 5’CCAACACCACACCAAAGCTG 3’ |
| *NbPR1a* for RT-qPCR | *NbPR1a-q* F: 5’ CCGCCTTCCCTCAACTCAAC 3’  *NbPR1a-q* R: 5’ GCACAACCAAGACGTACTGAG 3’ |
| *NbLOX* for RT-qPCR | *NbLOX-q* F: 5’ AAAACCTATGCCTCAAGAAC 3’  *NbLOX-q* R: 5’ ACTGCTGCATAGGCTTTGG 3’ |
| *NbERF1* for RT-qPCR | *NbERF1-q* F: 5’ GCTCTTAACGTCGGATGGTC 3’  *NbERF1-q* R: 5’ AGCCAAACCCTAGCTCCATT 3’ |
| *NbHIN1* for RT-qPCR | *NbHIN1-q* F: 5’ CCAACTTGAACGGAGCCTATTA 3’  *NbHIN1-q* R: 5’ AGGCATCCAAAGAGACAACTAC 3’ |
| *NbWRKY7* for RT-qPCR | *NbWRKY7-q* F: 5’ CACAAGGGTACAAACAACACAG 3’  *NbWRKY7-q* R: 5’ GGTTGCATTTGGTTCATGTAAG 3’ |
| *NbPTI5* for RT-qPCR | *NbPTI5-q* F: 5’ CCTCCAAGTTTGAGCTCGGATAGT 3’  *NbPTI5-q* R: 5’ CCAAGAAATTCTCCATGCACTCTGTC 3’ |
| *NbEF1α* for RT-qPCR | *NbEF1α*-q F: 5’ TGGACACAGGGACTTCATCA 3’  *NbEF1α*-q R: 5’ CAAGGGTGAAAGCAAGCAAT 3’ |
